# Supplementary material for: Synthesis of structurally controlled hyperbranched polymers using a monomer having hierarchical reactivity
Source: Nat Commun. 2017 Nov 30;8:1863. doi: 10.1038/s41467-017-01838-0 (PMC5709361; doi:10.1038/s41467-017-01838-0)
Supplement: Supplementary file 1 — Supplementary Information [file 41467_2017_1838_MOESM1_ESM.pdf]

Supplementary Methods

Supplementary Note 1: Density functional theory (DFT) calculation. All calculations were performed with Gaussian 09 program using the B3LYP method with the LANL2DZ basis set for Te atom and the 6-31G(d, p) basis set for the other atoms[1].

Supplementary Note 2: General. All reaction conditions dealing with oxygen and moisture sensitive compounds were carried out in a dry reaction vessel under a nitrogen atmosphere. ^{1}H NMR (400 MHz) and ^{13}C NMR (128 MHz) spectra were measured for a $CDCl_3$ solution of a sample and are reported in ppm (δ) from internal tetramethylsilane for ^{1}H NMR and from the solvent peak for ^{13}C NMR. Infrared (IR) spectra are reported in cm^{-1}. High resolution mass spectra (HRMS) were obtained under electron ionization (EI) condition. Size exclusion chromatography (SEC) was performed on a machine equipped with two linearly connected polystyrene (PSt) mixed gel columns (Shodex LF-604) at 40 °C using refractive index (RI) detector and a multi-angle laser light scattering (MALLS) detector (Wyatt Technology, DAWN EOS). THF, $CHCl_3$, and DMF (containing 0.01 mol L^{-1} of LiBr) were used as an eluent. The SEC traces were calibrated against poly(methyl methacrylate) (PMMA) or polystyrene (PSt) standards. The d*n*/d*c* value used for the MALLS analyses was determined using an RI detector by measuring RIs of a sample with three different concentrations (Supplementary Figure 1). AFM sample was prepared by spin casting (5000 rpm for 30 seconds) a polymer solution on a freshly made mica surface. That polymer solution (0.001 mg/ml in $CHCl_3$) was prepared by filtration through a PTFE membrane (Millex® – LH 0.45 µm). A 500 W high pressure mercury lamp, and 6 W white LED with combination of neutral density filter (Sigma Koki) with the combination of a cutoff filter (Asahi Techno Glass) was used as light source.

Supplementary Note 3: Materials. Unless otherwise noted, chemicals obtained from commercial supplies were used as received. Methyl acrylate (MA) were washed with 5% aqueous NaOH solution and distilled over CaH_2 and were deaerated by passing through nitrogen gas. 2-(Dimethylamino)ethyl acrylate and *N*,*N*-dimethylacrylamide were distilled from CaH_2 and were deaerated by passing through nitrogen gas. 2,2'-Azobisisobutyronitrile (AIBN) was recrystallized from methanol and was dissolved in deaerated benzene (c = 0.83 mol L^{-1}) to prepare a stock solution. MeOH was distilled from $CaCl_2$ and deaerated by passing through nitrogen gas. Organotellurium chain transfer agents (CTAs) **9**, **12**, and **13**[2,3] and dimethyl ditelluride were prepared as reported[4].

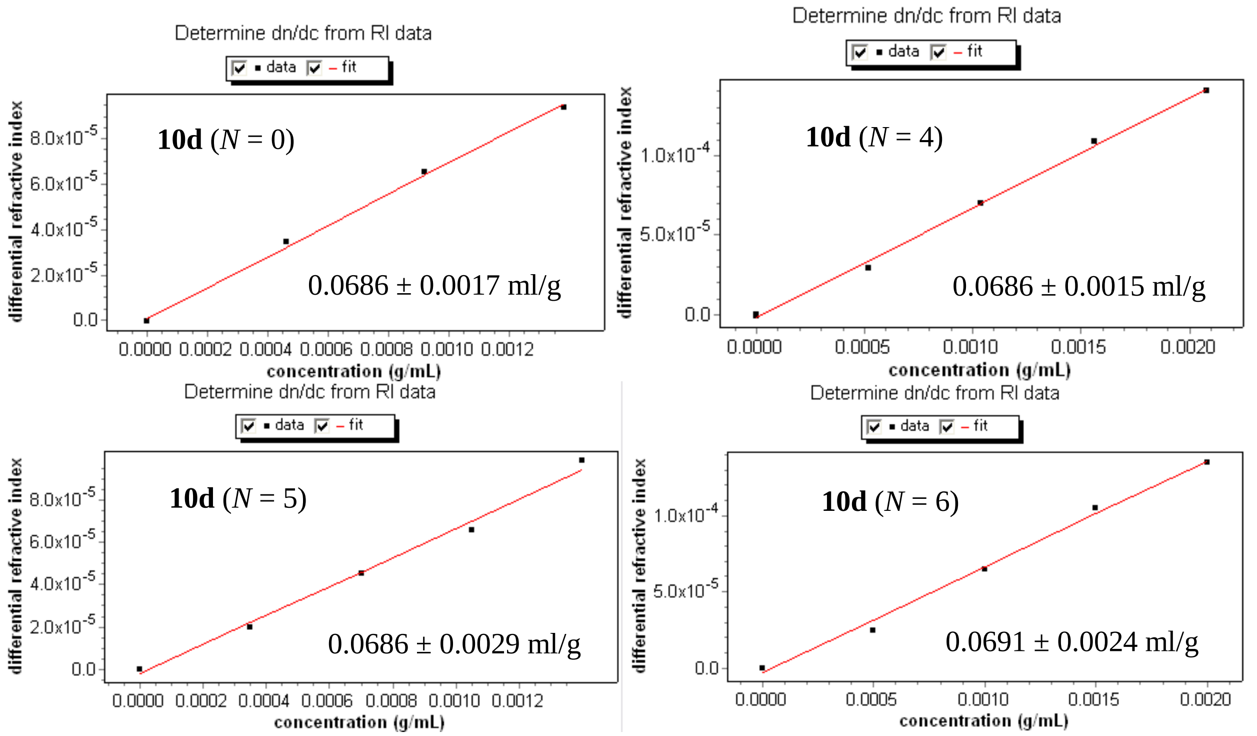

Supplementary Figure 1. Measurement of d*n*/d*c* value for **10d** with different dendritic generation (*N* = 0, 4, 5, and 6) for MALLS analyses.

Supplementary Note 4: Synthesis of 2-methyltellanylpropene (6a).

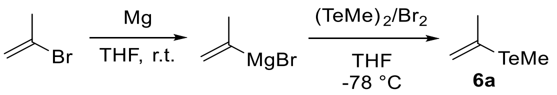

Magnesium (0.27 g, 11 mmol), an aliquot of I_2, and anhydrous THF (5 ml) were charged into a three-neck-flask equipped with a dripping funnel. 2-Bromo-propane (0.9 ml, 10 mmol) and anhydrous THF (20 ml) was charged to the funnel and were added dropwisely into the flask after color of I_2 was faded. The mixture was stirred at room temperature for 2 hours and the resulting Grignard reagent solution was cooled to -78 °C. To a separated two-neck flask containing dimethyl ditelluride (1.43 g, 5.0 mmol) and anhydrous THF (20 ml), bromine (0.26 ml, 5.0 mmol) was added at 0 °C. After 30 mins of stirring at 0 °C, the dark red liquid formed was added dropwisely into the Grignard reagent solution by a cannula at -78 °C. After stirring at this temperature for 30 mins, the mixture was pour into ice water (300 ml) with vigorous stirring. The aqueous phase was extracted with pentane (30 ml) for 5 times. The combined organic phase was washed with water (20 ml) for 10 times to remove THF. The organic residue was dried over $MgSO_4$ and filtered through a short plug of silica gel, and the solvent was removed by distillation at

normal pressure. The crude product was purified by distillation under vacuum (b.p 51-53 °C/33 mmHg) to give 0.79 g as a golden liquid (43 % yield).

^{1}H NMR ($CDCl_3$) 2.02 (s, Te satellite with J_{HTe} = 21.6 Hz, 3H), 2.19 (dd, J = 1.6, 1.2 Hz, Te satellite with J_{HTe} = 18.6 Hz, 3H), 5.24 (q, J = 1.1 Hz, Te satellite with J_{HTe} = 16.4 Hz, 1H), 5.78 (q, J = 1.6 Hz, Te satellite with J_{HTe} = 33.5 Hz, 1H), ^{13}C NMR ($CDCl_3$) -18.27 (Te*C*H_3), 29.15 (*C*H_3), 120.39 (H_2*C*=C), 121.42 (H_2C=*C*), IR (neat) 2947, 1605, 1431, 1369, 1219, 1165, 871, 833. Calcd for $[M]^+$, 185.9668, found 185.9685.

Supplementary Note 5: Synthesis of Me$_3$SiTeMe[5]. 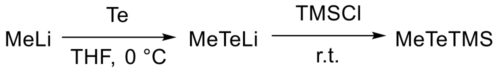

To a suspension of Te powder (6.37 g, 50 mmol) in anhydrous THF (20 ml) was added MeLi (1.19 mol L^{-1} in diethyl ether, 43.0 ml, 51 mmol) dropwisely through a dripping funnel at 0 °C. After string at room temperature for 30 mins, the solvent was partially removed in vacuum to leave ca. 7.5 ml of solution. Me_3SiCl (7.6 ml, 60 mmol) was added, and the reaction mixture was string at room temperature for 1 hour. The product and THF were collected in a liquid N_2 trap by vacuum transfer (6.5 mmHg) at room temperature to obtain a THF solution of $Me_3SiTeMe$ (9.0 ml, 1.18 mol L^{-1}, Yield 21%). The solution was used directly for the reduction without further purification.

^{1}H NMR ($CDCl_3$) 0.56 (s, 9H), 1.54 (s, Te satellite with J_{HTe} = 18.0 Hz, 3H).

Supplementary Note 6: Synthesis of 10d (Run 1). A solution of **9** (3.5 µl, 0.02 mmol), **6a** (6.5 µl, 0.06 mmol), MA (0.9 ml, 10 mmol), and AIBN (24 µl in benzene, 0.004 mmol) was heated at 60 °C in the dark for 24 hours. The polymerization was quenched by putting the reaction vessel in a freezer at -20 °C for 5 mins. Conversion of **6a** (>99%) and MA (95%) was determined from ^{1}H NMR spectroscopy. The crude mixture was dissolved in anhydrous THF (5 ml), and CH_3OH (35 µl, 0.86 mmol) and $Me_3SiTeMe$ (1.18 mol L^{-1} in THF, 80 µl, 0.094 mmol) were added at room temperature. The resulting solution was stirred for 1 hour at room temperature. The polymer sample was withdrawn and was analyzed by the SEC-MALLS ($M_{n(SEC)} = 4.21 \times 10^4$ g/mol, PDI = 1.55, $M_{n(MALLS)} = 5.37 \times 10^4$ g/mol).

Supplementary Note 7: Synthesis of 10d (Run 2). A solution of **9** (3.5 µl, 0.02 mmol), **6a** (15.5 µl, 0.14 mmol), MA (0.9 ml, 10 mmol), and AIBN (24 µl in benzene, 0.004 mmol) was heated at 60 °C in the dark for 30 hours. The polymerization was quenched by putting the reaction vessel in a freezer at -20 °C for 5 mins. Conversion of **6a** (>99%) and MA (95%) was determined from ^{1}H NMR spectroscopy. The crude mixture was dissolved in anhydrous THF (5 ml), and CH_3OH (70 µl, 1.7 mmol) and $Me_3SiTeMe$ (1.18 mol L^{-1} in THF, 160 µl, 0.19 mmol) were added at room temperature. The resulting solution was

stirred for 1 hour at room temperature. The polymer sample was withdrawn and was analyzed by the SEC-MALLS ($M_{n(SEC)}$ = 3.27 × 10^4 g/mol, PDI = 1.62, $M_{n(MALLS)}$ = 5.35 × 10^4 g/mol).

Supplementary Note 8: Synthesis of 10d (Run 3). A solution of **9** (3.5 µl, 0.02 mmol), **6a** (33.5 µl, 0.30 mmol), MA (0.9 ml, 10 mmol), and AIBN (24 µl in benzene, 0.004 mmol) was heated at 60 °C in the dark for 43 hours. The polymerization was quenched by putting the reaction vessel in a freezer at -20 °C for 5 mins. Conversion of **6a** (>99%) and MA (90%) was determined from ^{1}H NMR spectroscopy. The crude mixture was dissolved in anhydrous THF (5 ml), and CH_3OH (140 µl, 3.4 mmol) and $Me_3SiTeMe$ (1.18 mol L^{-1} in THF, 320 µl, 0.38 mmol) was added at room temperature. The resulting solution was stirred for 1 hour at room temperature. The polymer sample was withdrawn and was analyzed by the SEC-MALLS ($M_{n(SEC)}$ = 2.56 × 10^4 g/mol, PDI = 1.71, $M_{n(MALLS)}$ = 5.73 × 10^4 g/mol).

Supplementary Note 9: Synthesis of 10d (Run 4). A solution of **9** (3.5 µl, 0.02 mmol), **6a** (69 µl, 0.62 mmol), MA (0.9 ml, 10 mmol), and AIBN (24 µl in benzene, 0.004 mmol) was heated at 60 °C in the dark for 24 hours. A solution of AIBN (24 µl in benzene, 0.004 mmol) was added and the resulting mixture was further heated at 60 °C for 60 hours. The polymerization was quenched by putting the reaction vessel in a freezer at -20 °C for 5 mins. Conversion of **6a** (>99%) and MA (90%) was determined from ^{1}H NMR spectroscopy. The crude mixture was dissolved in anhydrous THF (5 ml), and CH_3OH (280 µl, 6.9 mmol) and $Me_3SiTeMe$ (1.18 mol L^{-1} in THF, 640 µl, 0.76 mmol) were added at room temperature. The resulting solution was stirred for 1 hour at room temperature. The polymer sample was withdrawn and was analyzed by the SEC-MALLS ($M_{n(SEC)}$ = 1.71 × 10^4 g/mol, PDI = 1.97, $M_{n(MALLS)}$ = 5.60 × 10^4 g/mol).

Supplementary Note 10: Synthesis of 10d (Run 5). A solution of **9** (3.5 µl, 0.02 mmol), **6a** (140 µl, 1.26 mmol), MA (0.9 ml, 10 mmol), AIBN (24 µl in benzene, 0.004 mmol), and 1,4-dimethoxybenzene (3.0 mg, 0. 022 mmol, an internal standard) was heated at 60 °C in the dark for 84 hours. A solution of AIBN (24 µl in benzene, 0.004 mmol) was added and the resulting mixture was further heated at 60 °C for 36 hours. The polymerization was quenched by putting the reaction vessel in a freezer at -20 °C for 5 mins. Conversion of **6a** (95%) and MA (74%) was determined from ^{1}H NMR spectroscopy of crude mixture. The crude mixture was dissolved in anhydrous THF (5 ml), and CH_3OH (0.8 ml, 20 mmol) and Me_3-SiTeMe (3.2 mol L^{-1} in THF, 0.5 ml, 1.6 mmol) were added at room temperature. The resulting solution was stirred for 1 hour at room temperature and was precipitated in 100 ml hexane twice. The resulting milky solution was centrifuged to obtain 0.46 g of colorless product. The resulting polymer was analyzed by the SEC-MALLS ($M_{n(SEC)}$ = 0.98 × 10^4 g/mol, PDI = 1.99, $M_{n(MALLS)}$ = 5.39 × 10^4 g/mol).

Supplementary Note 11: Synthesis of linear PMA (Run 6). A solution of **9** (3.5 µl, 0.02 mmol), MA (0.9 ml, 10 mmol), and AIBN (24 µl in benzene, 0.004 mmol) was heated at 60 °C for 2 hours. The polymerization was quenched by put the reaction vessel in a freezer at -20 °C for 5 mins. Conversion of MA (90%) was determined from ^{1}H NMR spectroscopy of crude mixture. The crude mixture was dissolved in anhydrous THF (5 ml), then Bu_3SnH (25 µl, 0.1 mmol) was added at room temperature. The resulting solution was stirred for 1 hour at 60 °C under the irradiation of 6W LED light and was precipitated in 100 ml hexane for twice. The resulting milky solution was centrifuged to obtain 0.83 g of colorless product. The resulting polymer was analyzed by the SEC-MALLS ($M_{n(SEC)}$ = 3.96 × 10^4 g/mol, PDI = 1.12, $M_{n(MALLS)}$ = 3.72 × 10^4 g/mol).

Supplementary Note 12: Synthesis of 10d (Run 7). A solution of **9** (8.8 µl, 0.05 mmol), **6a** (83.5 µl, 0.75 mmol), MA (0.45 ml, 5.0 mmol), and AIBN (60 µl in benzene, 0.01 mmol) was heated at 60°C in the dark for 66 hours. The polymerization was quenched by putting the reaction vessel in a freezer at -20 °C for 5 mins. Conversion of **6a** (>99%) and MA (92%) was determined from ^{1}H NMR spectroscopy of crude mixture. The crude mixture was dissolved in anhydrous THF (5 ml), and CH_3OH (0.5 ml, 20 mmol) and $Me_3SiTeMe$ (3.2 mol L^{-1} in THF, 0.3 ml, 0.32 mmol) were added at room temperature. The solution was stirred for 1 hour at room temperature. The polymer solution was precipitated in 100 ml hexane for twice. The resulting milky solution was centrifuged to obtain 0.17 g of colorless product. The resulting polymer was analyzed by the SEC-MALLS ($M_{n(SEC)}$ = 0.56 × 10^4 g/mol, PDI = 1.40, $M_{n(MALLS)}$ = 0.91 × 10^4 g/mol).

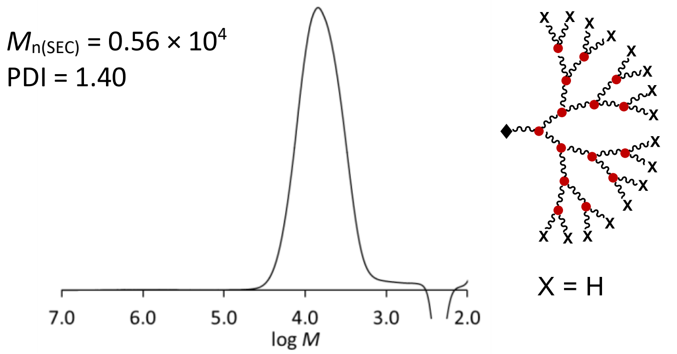

Supplementary Figure 2. SEC trace of hyperbranched PMA.

Supplementary Note 13: Synthesis of 10d (Run 8). A solution of **9** (3.5 µl, 0.02 mmol), **6a** (33.5 µl, 0.30 mmol), MA (0.45 ml, 5.0 mmol), and AIBN (24 µl in benzene, 0.004 mmol) was heated at 60 °C in the dark for 66 hours. The polymerization was quenched by put the reaction vessel in a freezer at -20 °C

for 5 mins. Conversion of **6a** (>99%) and MA (92%) was determined from ^{1}H NMR spectroscopy of crude mixture. The crude mixture was dissolved in anhydrous THF (5 ml), and CH_3OH (0.5 ml, 20 mmol) and $Me_3SiTeMe$ (3.2 mol L^{-1} in THF, 0.1 ml, 0.32 mmol) were added at room temperature. The resulting solution was stirred for 1 hour at room temperature and was precipitated in 100 ml hexane for twice. The resulting milky solution was centrifuged to obtain 0.31 g of colorless product. The resulting polymer was analyzed by the SEC-MALLS ($M_{n(SEC)}$ = 1.20 × 10^4 g/mol, PDI = 1.87, $M_{n(MALLS)}$ = 2.40 × 10^4 g/mol).

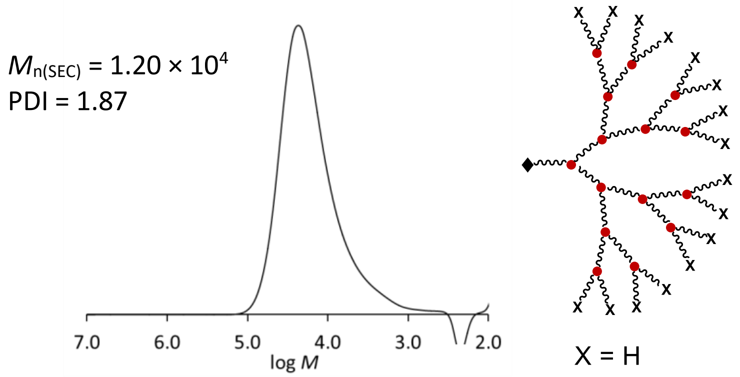

Supplementary Figure 3. SEC trace of hyperbranched PMA.

Supplementary Note 14: Synthesis of 10d (Run 9). A solution of **9** (1.8 µl, 0.01 mmol), **6a** (16.5 µl, 0.15 mmol), MA (1.8 ml, 20 mmol), and AIBN (12 µl in benzene, 0.002 mmol) was heated at 60 °C in the dark for 80 hours. The polymerization was quenched by putting the reaction vessel in a freezer at -20 °C for 5 mins. Conversion of **5a** (>99%) and MA (90%) was determined from ^{1}H NMR spectroscopy of crude mixture. The crude mixture was dissolved in anhydrous THF (20 ml), and CH_3OH (70 µl, 1.8 mmol) and $Me_3SiTeMe$ (1.18 mol L^{-1} in THF, 160 µl, 0.19 mmol) were added at room temperature. The resulting solution was stirred for 1 hour at room temperature and was precipitated in 200 ml hexane three times. The resulting milky solution was centrifuged to obtain 1.27 g of polymer product. The resulting polymer was analyzed by the SEC-MALLS ($M_{n(SEC)}$ = 7.47 × 10^4 g/mol, PDI = 1.91, $M_{n(MALLS)}$ = 16.21 × 10^4 g/mol).

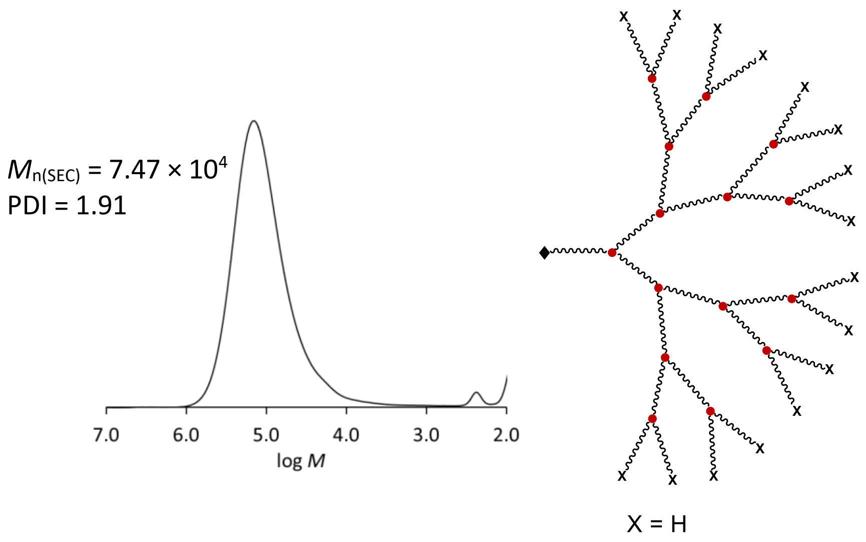

Supplementary Figure 4. SEC trace of hyperbranched PMA.

Supplementary Note 15: Synthesis of 10d (Run 10). A solution of **9** (1.8 µl, 0.01 mmol), **6a** (140 µl, 1.26 mmol), MA (1.8 ml, 20 mmol), and AIBN (12 µl in benzene, 0.002 mmol) was heated at 60 °C in the dark for 11 hours. A solution of AIBN (12 µl in benzene, 0.002 mmol) was added and the resulting mixture was further heated at 60 °C for 60 hours. Another solution of AIBN (12 µl in benzene, 0.002 mmol) was added and the resulting mixture was further heated at 60 °C for 61 hours. The polymerization was quenched by putting the reaction vessel in a freezer at -20 °C for 5 mins. Conversion of **6a** (>99%) and MA (90%) was determined from ^{1}H NMR spectroscopy of crude mixture. The crude mixture was dissolved in anhydrous THF (20 ml), and CH_3OH (1 ml, 25 mmol) and $Me_3SiTeMe$ (1.18 mol L^{-1} in THF, 1.25 ml, 1.5 mmol) were added at room temperature. The resulting solution was stirred for 1 hour at room temperature and was precipitated in 200 ml hexane three times. The resulting milky solution was centrifuged to obtain 1.36 g of polymer product. The resulting polymer was analyzed by the SEC-MALLS ($M_{n(SEC)}$ = 5.69 × 10^4 g/mol, PDI = 2.08, $M_{n(MALLS)}$ = 17.65 × 10^4 g/mol).

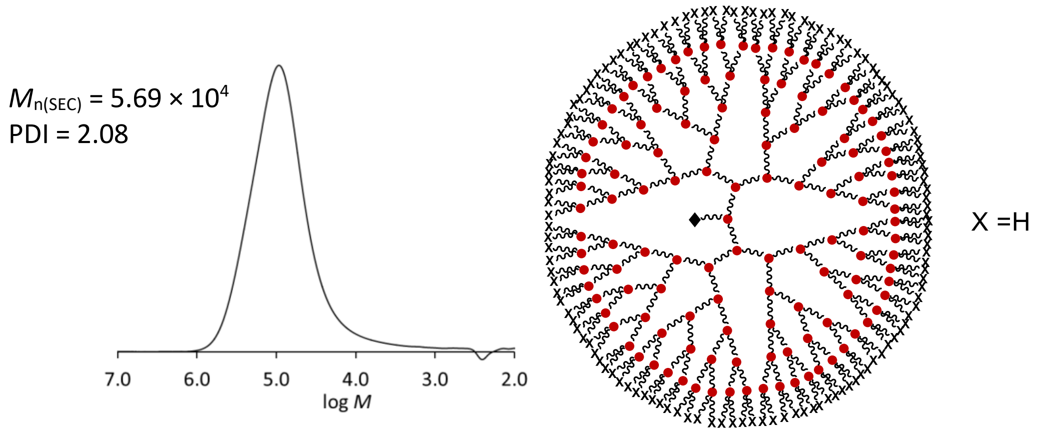

Supplementary Figure 5. SEC trace of hyperbranched PMA.

Supplementary Note 16: Synthesis of 10d (Run 11). A solution of **9** (1.8 µl, 0.01 mmol), **6b** (16.5 µl, 0.15 mmol), MA (0.45 ml, 5.0 mmol), and AIBN (12 µl in benzene, 0.002 mmol) was heated at 60 °C in the dark for 72 hours. The polymerization was stopped by putting the reaction vessel in a freezer at -20 °C for 5 mins. Conversion of **6b** (>99%) and MA (84%) was determined from ^{1}H NMR spectroscopy. $M_{n(SEC)}$ (2.18×10^4 g/mol) and PDI (1.80) were determined by SEC using NMR sample. The crude mixture was dissolved with anhydrous THF (5 ml), and anhydrous CH_3OH (70 µl, 1.7 mmol) and $Me_3SiTeMe$ (1.18 mol L^{-1} in THF, 160 µl, 0.19 mmol) and stirred for 1 hour at room temperature. The resulting solution was precipitated in 100 ml hexane for twice. The resulting milky solution was centrifuged to obtain 0.41 g of colorless polymer.

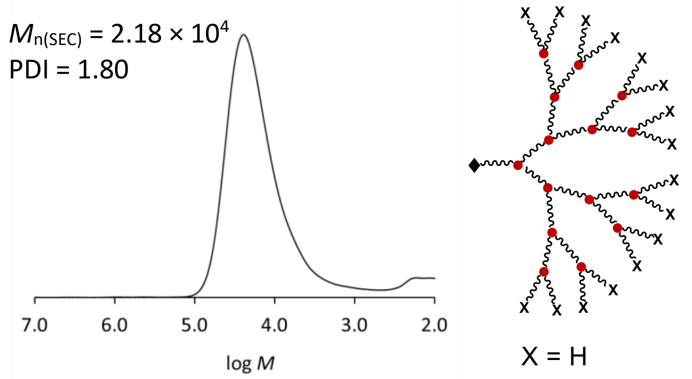

Supplementary Figure 6. SEC trace of hyperbranched PMA.

Supplementary Note 17: Synthesis of 10d* and 10d*-D (Run 12). A solution of **9** (1.8 µl, 0.01 mmol), **6b*** (16.5 µl, 0.15 mmol), MA (0.45 ml, 5.0 mmol), and AIBN (12 µl in benzene, 0.002 mmol) was heated at 60 °C in the dark for 72 hours. The polymerization was quenched by putting the reaction vessel in a freezer at -20 °C for 5 mins. Conversion of **6b*** (>99%) and MA (85%) was determined

from ^{1}H NMR spectroscopy. $M_{n(SEC)}$ (1.89 × 10^4 g/mol) and PDI (1.73) were determined by SEC using NMR sample. The crude mixture was dissolved with anhydrous THF (5 ml) and separated into two fractions. One of the fraction was added CH_3OD (100 µl, 2.5 mmol) and $Me_3SiTeMe$ (1.18 mol L^{-1} in THF, 100 µl, 0.12 mmol), and the resulting solution was stirred for 1 hour at room temperature. The polymer solution was precipitated in 100 ml hexane for twice. The resulting milky solution was centrifuged to obtain 0.23 g of colorless polymer. This sample was subjected to ^{2}H and ^{13}C NMR analyses. The other fraction was added CH_3OH (100 µl, 2.5 mmol) and $Me_3SiTeMe$ (1.18 mol L^{-1} in THF, 100 µl, 0.12 mmol), and the resulting solution was stirred for 1 hour at room temperature. The polymer solution was precipitated in 100 ml hexane for twice. The resulting milky solution was centrifuged to obtain 0.21 g of colorless polymer. This sample was subjected to ^{13}C NMR analysis.

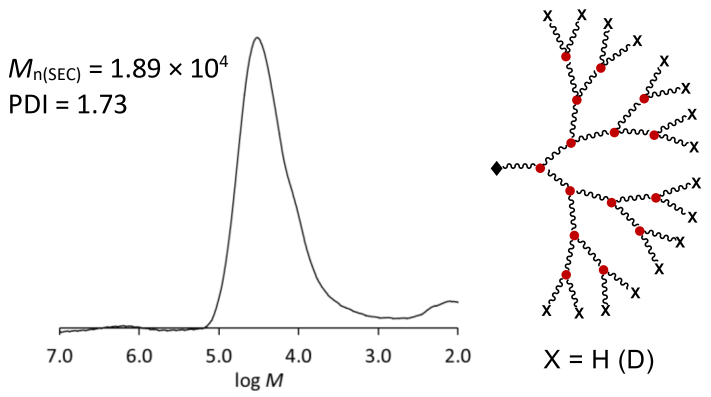

Supplementary Figure 7. SEC trace of hyperbranched PMA.

Supplementary Note 18: Synthesis of linear-*co*-branched PMA (Run 13). A solution of **9** (3.5 µl, 0.02 mmol) and MA (0.9 ml, 10 mmol) was heated at 60 °C under the irradiation of 6W LED light for 15 mins. The polymerization was quenched by putting the reaction vessel in a freezer at -20 °C for 5 mins. Conversion of MA (32%) was determined from ^{1}H NMR spectroscopy. $M_{n(SEC)}$ (1.51 × 10^4 g/mol) and PDI (1.12) were determined by SEC. To the polymer solution was added **6a** (33.5 µl, 0.3 mmol) and AIBN (24 µl in benzene, 0.004 mmol), and the resulting mixture was heated at 60 °C in the dark for 43 hours. Conversion of **6a** (>99%) and MA (88%) was determined from ^{1}H NMR spectroscopy. The crude mixture was dissolved in anhydrous THF (5 ml), and anhydrous CH_3OH (140 µl, 3.4 mmol) and Me_3-SiTeMe (3.2 mol L^{-1} in THF, 320 µl, 0.64 mmol) were added at room temperature. The resulting solution was stirred for 1 hour at room temperature, and the polymer sample was withdrawn and was analyzed by SEC-MALLS ($M_{n(SEC)}$ = 2.95 × 10^4 g/mol, PDI = 1.49, $M_{n(MALLS)}$ = 5.42 × 10^4 g/mol).

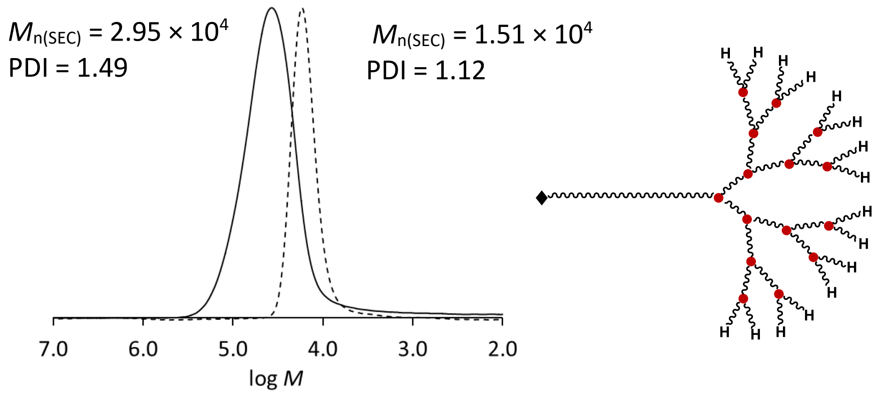

Supplementary Figure 8. SEC traces of macroinitiator **11** and linear-*block*-hyperbranched PMA.

Supplementary Note 19: Synthesis of dumbbell-shape PMA (Run 14). A solution of **12** (0.08 mol L^{-1} in DMF, 124 µl, 0.01 mmol), **6a** (33.5 µl, 0.3 mmol), MA (0.45 ml, 5.0 mmol), AIBN (24 µl in benzene, 0.004 mmol), and 1,4-dimethoxybenzene (3.1 mg, 0. 023 mmol, an internal standard) was heated at 60 °C in the dark for 12 hours. A solution of AIBN (24 µl in benzene, 0.004 mmol) was added and the resulting mixture was further heated at 60 °C for 68 hours. The polymerization was quenched by putting the reaction vessel in a freezer at -20 °C for 5 mins. Conversion of **6a** (>99%) and MA (97%) was determined from ^{1}H NMR spectroscopy. The crude mixture was dissolved in anhydrous THF (5 ml), and CH_3OH (140 µl, 3.4 mmol) and $Me_3SiTeMe$ (3.2 mol L^{-1} in THF, 320 µl, 0.64 mmol) were added at room temperature. The resulting solution was stirred for 1 hour at room temperature, and the polymer sample was withdrawn and was analyzed by SEC-MALLS ($M_{n(SEC)}$ = 1.74 × 10^4 g/mol, PDI = 1.89, $M_{n(MALLS)}$ = 4.67 × 10^4 g/mol).

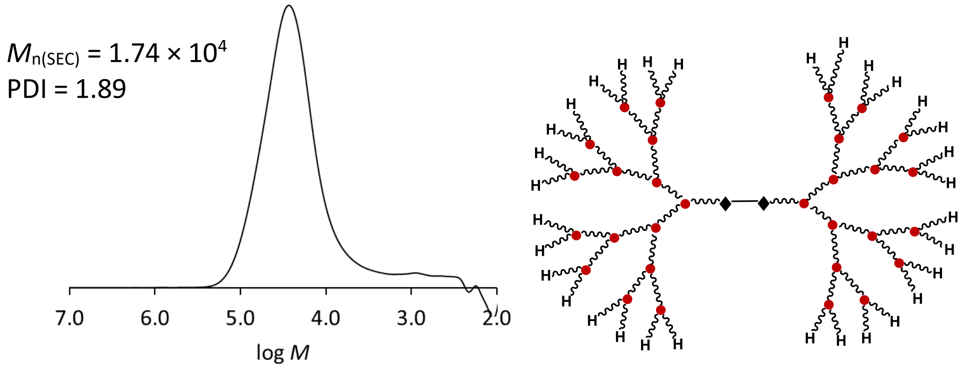

Supplementary Figure 9. SEC trace of dumbbell-shaped PMA.

Supplementary Note 20: Synthesis of clover-shape PMA (Run 15). A solution of **13** (0.06 mol L^{-1} in DMF, 166 µl, 0.01 mmol), **6a** (23.5 µl, 0.21 mmol), MA (0.45 ml, 5.0 mmol), and AIBN (12 µl in

benzene, 0.002 mmol) was heated at 60 °C in the dark for 72 hours. A solution of AIBN (24 μl in benzene, 0.004 mmol) was added and the resulting mixture was further heated at 60 °C for 96 hours. The polymerization was quenched by putting the reaction vessel in a freezer at -20 °C for 5 mins. Conversion of **6a** (>99%) and MA (84%) was determined from ^{1}H NMR spectroscopy. The crude mixture was dissolved in anhydrous THF (5 ml), and CH_3OH (100 μl, 2.5 mmol) and $Me_3SiTeMe$ (1.18 mol L^{-1} in THF, 210 μl, 0.25 mmol) were added at room temperature. The resulting solution was stirred for 1 hour at room temperature, and the polymer sample was withdrawn and was analyzed by SEC-MALLS ($M_{n(SEC)}$ = 1.73 × 10^4 g/mol, PDI = 1.44, $M_{n(MALLS)}$ = 5.66 × 10^4 g/mol).

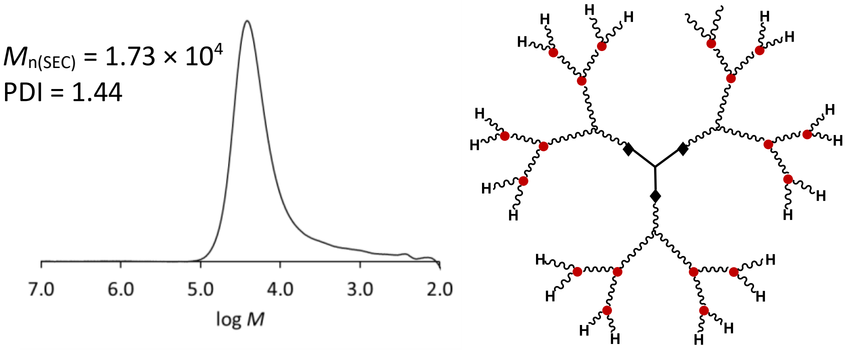

Supplementary Figure 10. SEC trace of clover-shaped PMA.

Supplementary Note 21: Synthesis of hyperbranched poly(2-(Dimethylamino)ethyl acrylate) (Run 18). A solution of **9** (1.8 μl, 0.01 mmol), **6a** (16.5 μl, 0.15 mmol), 2-(Dimethylamino)ethyl acrylate (0.76 ml, 5.0 mmol), AIBN (12 μl in benzene, 0.002 mmol) was heated at 60 °C in the dark for 84 hours. A solution of AIBN (12 μl in benzene, 0.002 mmol) was added and the resulting mixture was further heated at 60 °C for 72 hours. The polymerization was quenched by putting the reaction vessel in a freezer at -20 °C for 5 mins. Conversion of **6a** (>99%) and MA (71%) was determined from ^{1}H NMR spectroscopy of crude mixture. The crude mixture was dissolved in anhydrous THF (5 ml), and Bu_3SnH (80 μl, 0.3 mmol) was added at room temperature. The resulting solution was stirred for 2 hour at 60 °C with the irradiation of 6W LED light. The polymer sample was withdrawn and was analyzed by the SEC ($M_{n(SEC)}$ = 2.51 × 10^4 g/mol, PDI = 1.85).

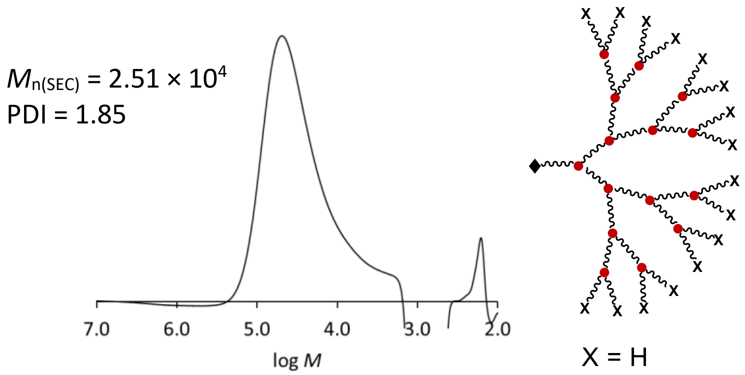

Supplementary Figure 11. SEC trace of hyperbranched poly(2-(Dimethylamino)ethyl acrylate).

Supplementary Note 22: Synthesis of hyperbranched poly(*N*,*N*-dimethylacrylamide) (Run 17). A solution of **9** (1.8 µl, 0.01 mmol), **6a** (16.5 µl, 0.15 mmol), *N*,*N*-dimethylacrylamide (0.51 ml, 5.0 mmol), AIBN (12 µl in benzene, 0.002 mmol) was heated at 60 °C in the dark for 7 hours. The polymerization was quenched by putting the reaction vessel in a freezer at -20 °C for 5 mins. Conversion of **6a** (81%) and MA (86%) was determined from ^{1}H NMR spectroscopy of crude mixture. The crude mixture was dissolved in anhydrous THF (5 ml), and Bu_3SnH (80 µl, 0.3 mmol) was added at room temperature. The resulting solution was stirred for 3 hours at 60 °C with the irradiation of 6W LED light. The polymer sample was withdrawn and was analyzed by the SEC ($M_{n(SEC)}$ = 2.02 × 10^4 g/mol, PDI = 1.45).

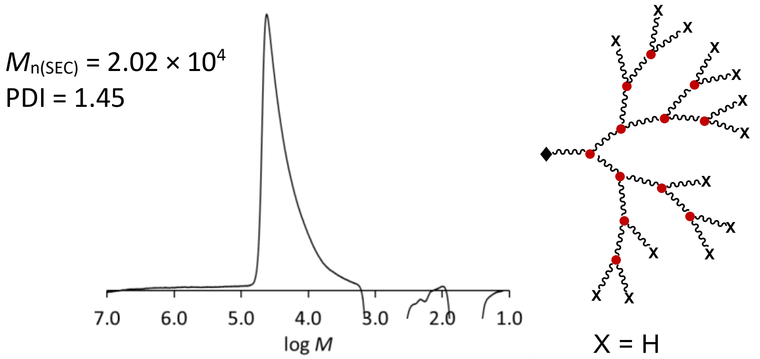

Supplementary Figure 12. SEC trace of hyperbranched poly(*N*,*N*-dimethylacrylamide).

Supplementary Note 23: Synthesis of 10e (Run 16). A solution of **9** (3.5 µl, 0.02 mmol), **6a** (33.5 µl, 0.3 mmol), MA (0.9 ml, 10 mmol), and AIBN (24 µl in benzene, 0.004 mmol) was heated at 60 °C in the dark for 56 hours. The polymerization was quenched by putting the reaction vessel in a freezer at -20 °C for 5 mins. Conversion of **6a** (>99%) and MA (80%) was determined from ^{1}H NMR spectroscopy of crude mixture. The crude mixture was vacuumed for 1.5 hours for the removal of residual monomer and then dissolved in anhydrous THF (5 ml). 4-Amino-2,2,6,6-tetramethylpiperidine-1-oxyl (69.3 mg, 0.4

mmol) were then added at room temperature. The resulting solution was irradiated with 500 W high pressure mercury lamp through 390 nm cutoff filter at 25 °C for 6 hours. The resulting solution was filtered through a plug of celite and then precipitated in hexane (100 ml). The resulting milky solution was centrifuged to obtain 0.87 g of polymer product. The resulting polymer was analyzed by the SEC ($M_{n(SEC)}$ = 1.34 × 10^4 g/mol, PDI = 2.12). The efficiency of end group transformation (>99%) was determined by ^{1}H NMR.

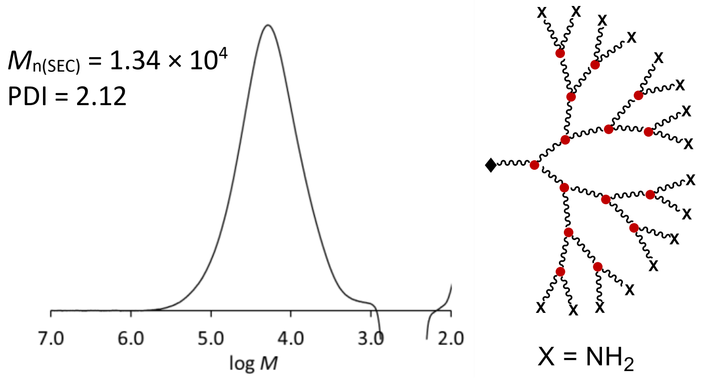

Supplementary Figure 13. SEC trace of *ω*-ends functionalized hyperbranched PMA.

Supplementary Note 24: Synthesis of 10d-D. A solution of **9** (3.5 μl, 0.02 mmol), **6a** (69 μl, 0.62 mmol), MA (0.9 ml, 10 mmol), and AIBN (24 μl in benzene, 0.004 mmol) was heated at 60 °C in the dark for 84 hours. A solution of AIBN (24 μl in benzene, 0.004 mmol) was added and the resulting mixture was further heated at 60 °C for 16 hours. The polymerization was quenched by putting the reaction vessel in a freezer at -20 °C for 5 mins. Conversion of **6a** (>99%) and MA (93%) was determined from ^{1}H NMR spectroscopy. The crude mixture was dissolved in anhydrous THF (5 ml), and CH_3OD (0.6 ml, 14.7 mmol) and $Me_3SiTeMe$ (3.2 mol L^{-1} in THF, 0.3 ml, 0.96 mmol) were added at room temperature. The resulting solution was stirred for 1 hour at room temperature. The polymer solution was precipitated in 100 ml hexane for twice. The resulting milky solution was centrifuged to obtain 0.68 g of colorless product. The resulting polymer was analyzed by the SEC-MALLS ($M_{n(SEC)}$ = 1.91 × 10^4 g/mol, PDI = 1.97, $M_{n(MALLS)}$ = 6.04 × 10^4 g/mol).

Supplementary Note 25: Synthesis of 6b.

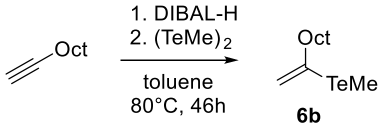

Diisobutylaluminum hydride (DIBAL-H) (1.0 mol L^{-1} in toluene, 3.5 ml, 5.3 mmol) was added to $(TeMe)_2$ (0.72 g, 2.5 mmol) at room temperature. The mixture was heated at 80°C for 30 mins, and the color of the solution turned to a light yellow. This solution was added to decayne (2.5 ml, 13 mmol) by

cannula. The reaction mixture was heated at 80 °C for 46 hour and was quenched by an unaerated aqueous solution of sodium tartrate (0.12 mol L^{-1} solution, 50 mL, 5.9 mmol). The mixture was extracted by hexane (20 ml) for five times. The combined organic phase was dried with $MgSO_4$, and the solvent and unreacted decayne were removed under vacuum. The crude product was purified by preparative SEC to obtained 0.58 g (48% yield) of **6b** as golden oil.

^{1}H NMR ($CDCl_3$) 0.88 (distorted t, J = 6.7 Hz, 3H), 1.28 (br s, 10H), 1.43-1.55 (m, 2H), 2.00 (s, Te satellite with J_{HTe} = 22.1 Hz, 3H), 2.31 (br t, J_{HH} = 7.8 Hz, 2H), 5.23 (s, Te satellite with J_{HTe} = 14.6 Hz, 1H), 5.82 (t, J_{HH} = 1.4 Hz, Te satellite with J_{HTe} = 28.4 Hz, 1H), ^{13}C NMR ($CDCl_3$) -18.35 (Te*C*H_3), 14.11 (CH_2*C*H_3), 22.67 (*C*H_2), 28.80 (*C*H_2), 29.25 (*C*H_2), 29.40 (*C*H_2), 29.64 (*C*H_2), 31.87 (*C*H_2), 42.19 (C(TeCH_3)*C*H_2), 120.07 (*C*H_2=C), 128.39 (*C*), IR (neat) 2924, 1601, 1462, 1219, 868, 833. HRMS *(m/z)*: $[M]^+$ calcd for $C_{11}H_{22}Te$, 284.0784, found 284.0777.

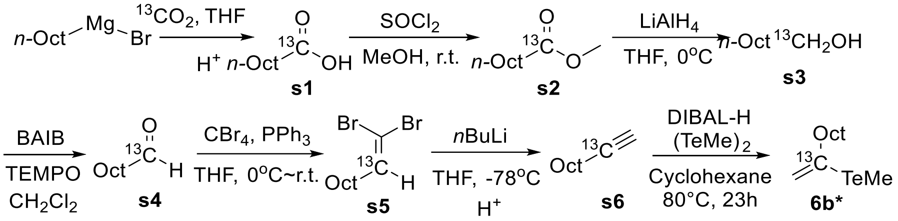

Supplementary Figure 14. Synthetic route of **6b***.

Supplementary Note 26: Synthesis of s1. A solution of 1-bromooctane (8.8 ml, 51 mmol) in anhydrous THF (30 ml) was added dropwisely to with a mixture of Mg (1.28 g, 53 mmol) and an aliquot of I_2 in anhydrous THF (20 ml), and the resulting mixture was stirring for 1 hour at room temperature. The flask was connected to a trap, which was cooled by liquid N_2, and $^{13}CO_2$ (26 mmol, >99% of isotropic purity) was introduced to the Grignard reagent solution. Then, the trap was heated to room temperature. The mixture was then quenched with aqueous HCl solution (1.0 mol L^{-1}, 100 ml) and was extracted with Et_2O (100 ml) for three times. The combined organic phase was dried over $MgSO_4$ and was filtrated. After removal of solvent under reduced pressure, the obtained light yellow oil (7.13 g) was used for next reaction without further purification.

^{1}H NMR ($CDCl_3$) 0.88 (distorted t, J = 6.1, 3H), 1.28 (br s, 10H), 1.63 (br s, 2H), 2.35 (dt, J_{HC} = 7.1, J_{HH} = 7.2 Hz, 2H).

Supplementary Note 27: Synthesis of s2. To a solution of crude **s1** in anhydrous MeOH (20 ml) was dropwisely added $SOCl_2$ (3.0 ml, 42 mmol) through a dripping funnel at 0 °C. After 3 hours, volatile materials were evacuated under vacuum and the resulting mixture was filtered through a plug of silica gel

with CH_2Cl_2 as an eluent. The solvent was evaporated under reduced pressure, and the resulting crude mixture was used for next reaction directly.

^{1}H NMR ($CDCl_3$) 0.88 (distorted t, J = 6.2, 3H), 1.19-1.41 (m, 10H), 1.50-1.75 (m, 2H), 2.30 (dt, J_{HC} = 7.3, J_{HH} = 7.4 Hz, 2H), 3.7 (distorted d, J_{HC} = 3.71 Hz, 3H).

Supplementary Note 28: Synthesis of s3. A solution of crude **s2** in anhydrous THF (30 ml) was slowly added to a suspension of $LiAlH_4$ (1.55 g, 41 mmol) in anhydrous THF (20 ml) through a dripping funnel at 0 °C. After 1 hour, the mixture was quenched with aqueous HCl (0.6 mol L^{-1} in ice water, 200 ml). The aqueous phase was extracted with 50 ml Et_2O for 4 times. The combined organic phase was washed with saturated aqueous $NaHCO_3$ solution (50 ml) twice and saturated aqueous NaCl solution (50 ml). The combined organic phase was dried over $MgSO_4$ and was evaporated to leave crude oil, which was used for next step without further purification.

^{1}H NMR ($CDCl_3$) 0.88 (distorted t, J = 6.2, 3H), 1.19-1.41 (m, 12H), 1.45-1.62 (m, 2H), 3.64 (dt, J_{HC} = 140.9, J_{HH} = 6.6 Hz, 2H).

Supplementary Note 29: Synthesis of s4. A mixture of crude **s3**, (diacetoxyiodo)benzene (9.67 g, 30 mmol) and 2,2,6,6-tetramethylpiperidinyloxy (0.21 g, 1.3 mmol) in CH_2Cl_2 (50 ml) was stirring for 1 hour at room temperature, and the mixture was washed with 50 ml saturated aqueous $NaHCO_3$ solution for three times. The organic phase was dried over $MgSO_4$ and solvents were evaporated under vacuum. The crude mixture was filtered through a plug of silica gel with hexane as an eluent to remove iodobenzene followed by CH_2Cl_2. The CH_2Cl_2 fraction was collected and removal of the solvent and distillation of the crude mixture under reduced pressure (b.p. 54~58°C /1.8 mmHg) to give **s4** as a colorless liquid. While the sample contained an impurity (3-hydroxypropyl acetate formed by ring opening of THF followed by acetylation), it was used to the next step without further purification.

^{1}H NMR ($CDCl_3$) 0.88 (distorted t, J = 6.3, 3H), 1.22-1.37 (m, 10H), 1.55-1.70 (m, 2H), 2.41 (br dt, J_{HC} = 6.8, J_{HH} = 6.9 Hz, 2H), 9.76 (br d, J_{HC} = 169.7, 1H).

Supplementary Note 30: Synthesis of s5. A solution of PPh_3 (10.3 g, 39 mmol) in anhydrous THF (50 ml) was added dropwisely to a solution of CBr_4 (6.67 g, 20 mmol) in anhydrous THF (20 ml) at 0 °C and the resulting mixture was stirred for 30 mins. A solution of **s4** in anhydrous THF (50 ml) was added dropwisely at 0 °C, and the mixture was stirred for 1 hour at this temperature. The solid was filtered and was washed with THF. The collected solution was evaporated, and the generated solid was filtered and was washed with hexane. The collected solution was filtered through a plug of silica gel with hexane as

an eluent. The solvent was removed under vacuum and the obtained crude oil was used for next reaction without further purification.

^{1}H NMR ($CDCl_3$) 0.88 (distorted t, J = 6.3, 3H), 1.27 (br s, 10H), 1.36-1.46 (m, 2H), 2.00-2.15 (m, 2H), 6.39 (dd, J_{HC} = 159.81, J_{HH} = 7.26 Hz, 1H).

Supplementary Note 31: Synthesis of s6. A solution of *n*-butyl lithium in hexane (32 ml, 1.58 mol L^{-1}, 49 mmol) was added dropwisely to a solution of crude **s5** in anhydrous THF (50 ml) at -78 °C. After 30 mins stirring at this temperature, the reaction mixture was quenched with saturated aqueous NH_4Cl (20 ml). The organic phase was dried over $MgSO_4$ and filtered through a plug of silica gel with hexane as an eluent. Removal of the solvent followed by distillation under reduced pressure (b.p. ~60 °C /20 mmHg) to give 0.45 g of **s6** (12% yield from 1-bromooctane) with >99% purity.

^{1}H NMR ($CDCl_3$) 0.88 (distorted t, J = 6.7 Hz, 3H), 1.21-1.34 (m, 8H), 1.34-1.44 (m, 2H), 1.48-1.58 (m, 2H), 1.94 (dt, J_{HC} = 49.2 Hz, J_{HH} = 2.7 Hz, 1H), 2.19 (ddt, J_{HC} = 7.3 Hz, J_{HH} = 7.1, 2.7 Hz, 2H), ^{13}C NMR ($CDCl_3$) 14.10 ($CH_2\mathit{C}H_3$), 22.66 ($\mathit{C}H_2CH_3$), 28.49 ($\mathit{C}H_2$), 28.76 ($\mathit{C}H_2$), 28.80 ($\mathit{C}H_2$), 29.08 ($\mathit{C}H_2$), 29.18 ($\mathit{C}H_2$), 31.83 ($\mathit{C}H_2$), 67.95 (d, J_{CC} = 171.5 Hz, $^{13}C{\equiv}\mathit{C}H$), 84.83 ($^{13}\mathit{C}{\equiv}CH$).

Supplementary Note 32: Synthesis of 6b*. DIBAL-H (1.2 ml, 1.0 mol L^{-1} in cyclohexane, 1.2 mmol) was added to $(TeMe)_2$ (0.10 g, 0.35 mmol), and the resulting solution was heated at 80°C for 30 mins until the mixture turned to light yellow solution. This solution was added to crude **s6** (0.31 g, 2.2 mmol) by cannula. The resulting mixture was stirring at 80 °C for 23 h and quenched by aqueous sodium tartrate solution (20 ml, 0.1 mol L^{-1}). The mixture was extracted by hexane (10 ml) for five times. The combined organic phase was dried over $MgSO_4$, and the solvent and unreacted decyne were removed under vacuum. The crude product was purified by preparative SEC to obtained 0.12 g of **5b*** and its regioisomer, 1-methyltellanyl-1-decene, as a 96:4 mixture in 65% combined yield as golden oil. A control experiment revealed the regioisomer had no reactivity for the copolymerization.

^{1}H NMR ($CDCl_3$) 0.88 (distorted t, J = 6.73 Hz, 3H), 1.21-1.35 (m, 10H), 1.43-1.55 (m, 2H), 2.00 (d, J_{HC} = 2.5 Hz, satellite signal with Te with J_{HTe} = 22.06 Hz, 3H), 2.31 (br dt, J_{HC} = 7.3 Hz, J_{HH} = 7.2 Hz, 2H), 5.23 (d, J_{HC} = 3.4 Hz, satellite signal with Te with J_{HTe} = 14.5 Hz, 1H), 5.82 (s, Te satellite with J_{HTe} = 28.4 Hz, 1H), ^{13}C NMR ($CDCl_3$) -18.35 ($Te\mathit{C}H_3$), 14.11 ($\mathit{C}H_3$), 22.66 ($\mathit{C}H_2$), 28.79 (d, J_{CC} = 2.5 Hz, $\mathit{C}H_2$), 29.24 ($\mathit{C}H_2$), 29.40 ($\mathit{C}H_2$), 29.65 (d, J_{CC} = 4.4 Hz, $\mathit{C}H_2$), 31.86 ($\mathit{C}H_2$), 42.17 (d, J_{CC} = 40.2 Hz, $CH_2{=}^{13}C(TeCH_3)\mathit{C}H_2$), 120.07 (d, J_{CC} = 70.9 Hz, $\mathit{C}H_2{=}^{13}C(TeCH_3)CH_2$), 128.39 ($CH_2{=}^{13}\mathit{C}(TeCH_3)CH_2$).

Results of SEC and NMR analyses

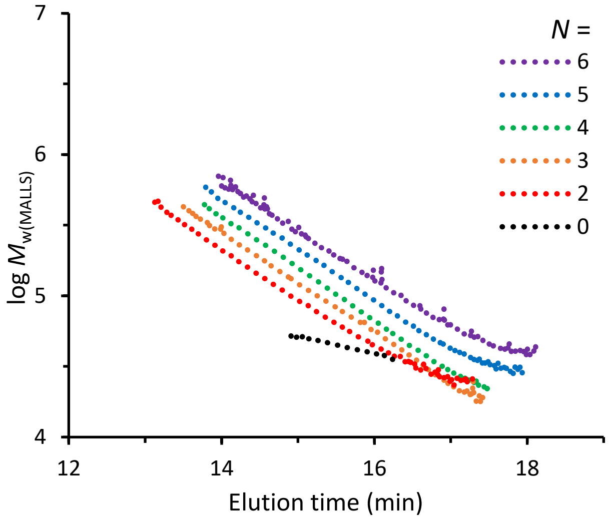

Supplementary Figure 15. Correlation between elution time and weight average molecular weight of **10a** (Table 1, runs 1-6) determined by MALLS.

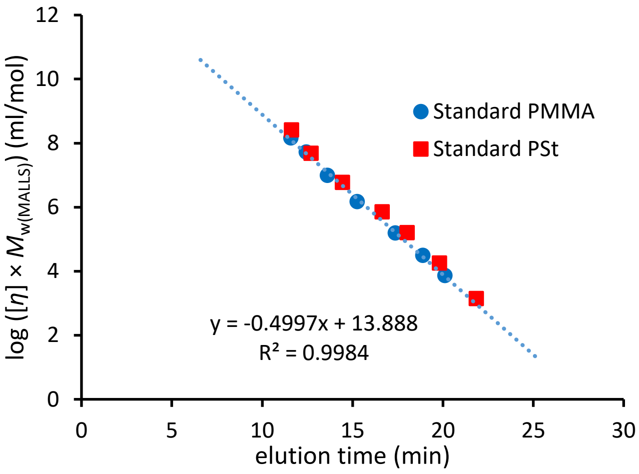

Supplementary Figure 16. Universal plot for SEC column calibrated from linear PMMA and PSt standard. The Mark-Hauwink constant is taken from the reported data[6-8].

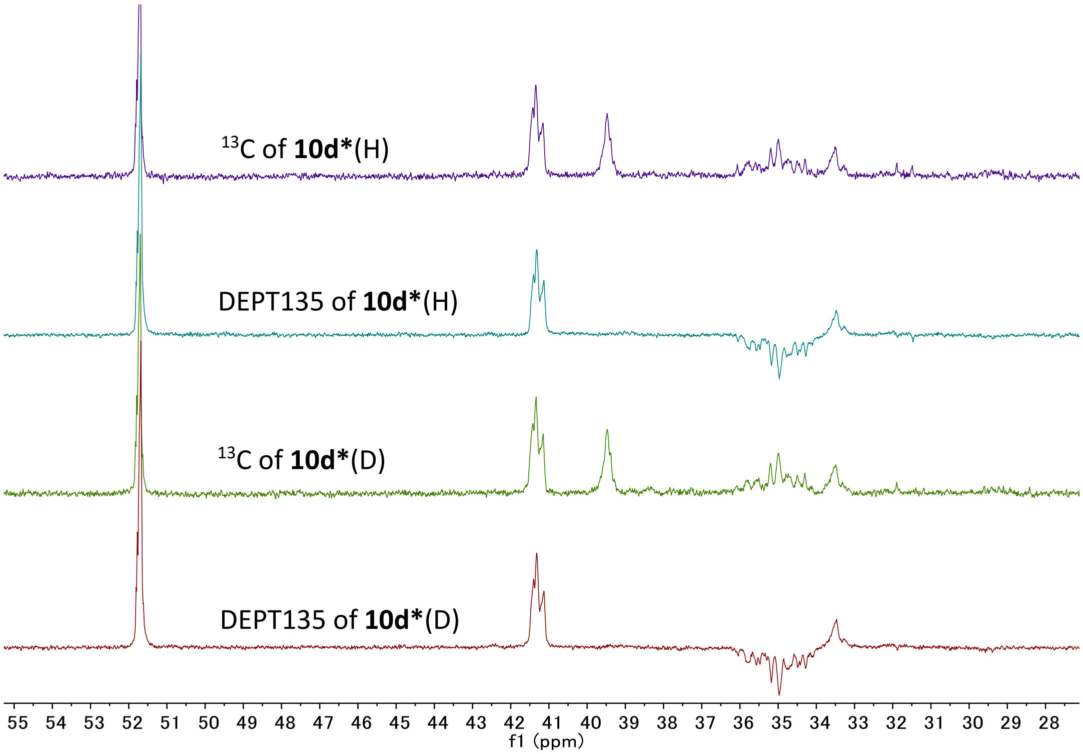

Supplementary Figure 17. ^{13}C NMR and DEPT 135° spectra of **10d***(D) after MeTeD reduction.

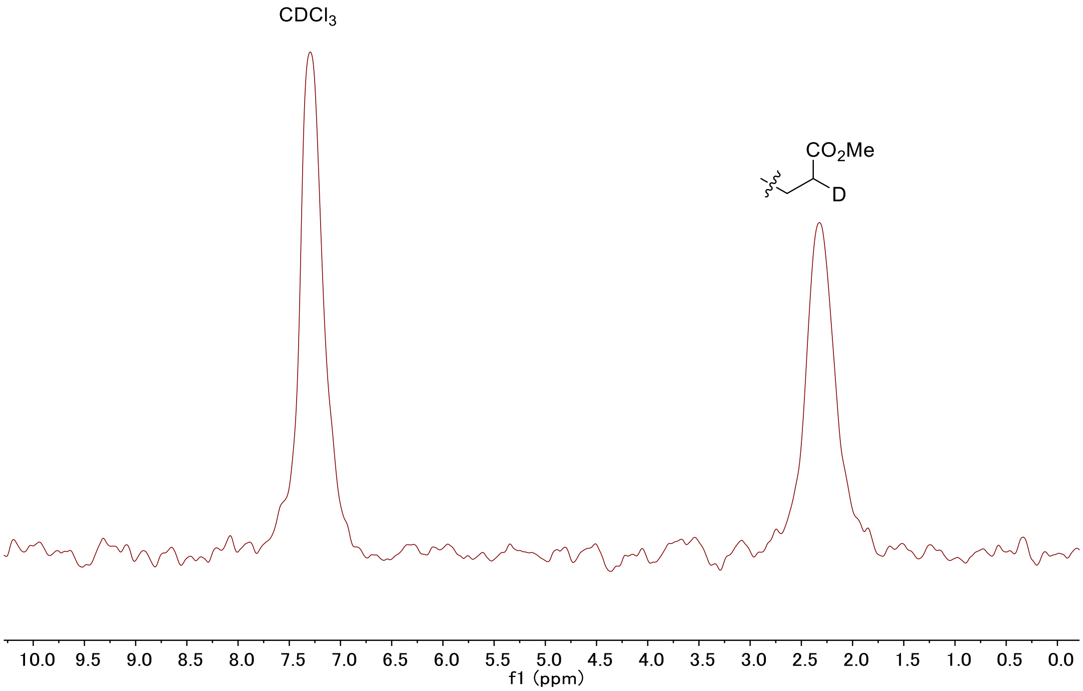

Supplementary Figure 18. ^{2}H NMR of **10d***(D).

Supplementary Table 1. Energies of the structure optimized compounds.

Compound	Hartree-Fock Energy (hartree)	Zero Point Energy (J/mol)	Total Energy (kJ/mol)
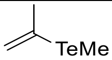	-165.2669456	281107.7	-433627.2579
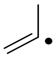	-117.23573617	172185.1	-307630.2402
•TeMe	-47.94144311	89260.2	-125780.9987
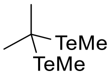	-213.85284139	412743.2	-561057.8919
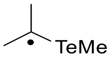	-165.84363448	306009.3	-435116.453
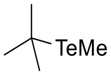	-205.81890506	416038.0	-539961.4972
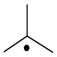	-157.81101068	306606.1	-414026.2024

Supplementary Table 2. Cartesian coordinate of the structurally optimized compounds

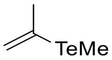

Atom	X	Y	Z
C	-1.6170408	1.4552728	0.0003165
H	-2.6858809	1.2357029	0.0004328
H	-1.3586442	2.0164315	-0.8973543
H	-1.3583925	2.0162928	0.8979997
Te	-0.5818515	-0.4477249	-0.0000634
C	1.4138031	0.3520550	-0.0001038
C	1.6777984	1.6597619	-0.0002326
H	0.9132624	2.4289316	-0.0003194
H	2.7104713	2.0048368	-0.0002672
C	2.4830862	-0.7099153	0.0002798
H	3.4772613	-0.2514569	-0.0011382
H	2.4068758	-1.3548952	0.8838359
H	2.4054451	-1.3571969	-0.8814542

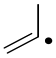

Atom	X	Y	Z
C	0.124999	-0.381722	-0.000131
C	1.346654	0.098547	0.000046
H	2.226178	-0.542734	0.000173
H	1.541578	1.177216	-0.000136
C	-1.273318	0.076677	0.000034
H	-1.332634	1.177258	-0.001027

H	-1.813015	-0.287222	-0.882159
H	-1.812121	-0.285532	0.883454

•TeMe

Atom	X	Y	Z
C	1.840059	0.004884	-0.000065
H	2.221070	1.024181	-0.004299
H	2.179156	-0.529311	0.889214
H	2.179543	-0.537193	-0.884368
Te	-0.338849	0.000250	-0.000003

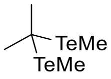

Atom	X	Y	Z
C	0.152053	1.912360	-1.252735
H	-0.728964	2.555017	-1.381320
H	0.270962	1.318794	-2.163808
H	1.030983	2.559513	-1.156258
C	0.000011	1.044256	-0.000284
C	-0.152051	1.913008	1.251716
H	-1.031004	2.560079	1.154908
H	-0.270935	1.319911	2.163097
H	0.728945	2.555761	1.379962
Te	1.821267	-0.189559	0.309693
Te	-1.821220	-0.189758	-0.309624
C	1.772254	-1.184411	-1.615993
H	0.759622	-1.554792	-1.784774
H	2.075664	-0.501781	-2.408959
H	2.467584	-2.023912	-1.565933
C	-1.772516	-1.183200	1.616799
H	-2.467885	-2.022700	1.567262
H	-0.759927	-1.553511	1.785986
H	-2.075995	-0.499972	2.409223

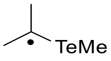

Atom	X	Y	Z
C	-1.368482	0.212698	0.162829
C	-2.405853	-0.857676	0.321807
H	-2.023406	-1.742311	0.840726

H	-3.257270	-0.472632	0.900599
H	-2.816305	-1.194357	-0.645659
C	-1.808724	1.564017	-0.308678
H	-2.704122	1.882302	0.243042
H	-1.045766	2.336312	-0.173697
H	-2.079436	1.560373	-1.378676
Te	0.629997	-0.424461	-0.088061
C	1.572853	1.476838	0.387733
H	2.643069	1.283902	0.477101
H	1.397922	2.202293	-0.405813
H	1.186685	1.840835	1.339378

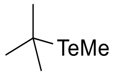

Atom	X	Y	Z
C	1.341824	0.134680	0.000001
C	1.667175	0.940554	1.261206
H	1.449209	0.374176	2.170665
H	2.736824	1.196049	1.266885
H	1.108848	1.880882	1.302229
Te	-0.827050	-0.432139	0.000002
C	1.667077	0.940919	-1.260996
H	2.736725	1.196420	-1.266687
H	1.449042	0.374801	-2.170601
H	1.108739	1.881253	-1.301706
C	-1.638627	1.584908	0.000001
H	-2.725492	1.485028	-0.000002
H	-1.327748	2.120625	0.896839
H	-1.327735	2.120643	-0.896822
C	2.104841	-1.196077	-0.000220
H	1.879485	-1.795367	-0.888347
H	3.185376	-0.997567	-0.000259
H	1.879592	-1.795605	0.887774

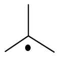

Atom	X	Y	Z
C	-0.000027	-0.000052	-0.144985
C	1.261029	-0.791002	0.014681
H	1.176196	-1.786578	-0.436704
H	2.119899	-0.282400	-0.439000

H	1.520558	-0.952165	1.077471
C	0.054574	1.487505	0.014678
H	0.064548	1.792609	1.077528
H	0.959181	1.911766	-0.436781
H	-0.815304	1.977266	-0.438803
C	-1.315606	-0.696497	0.014682
H	-1.584902	-0.840120	1.077537
H	-2.135243	-0.125299	-0.437005
H	-1.304761	-1.694806	-0.438579

NMR spectra of new compounds

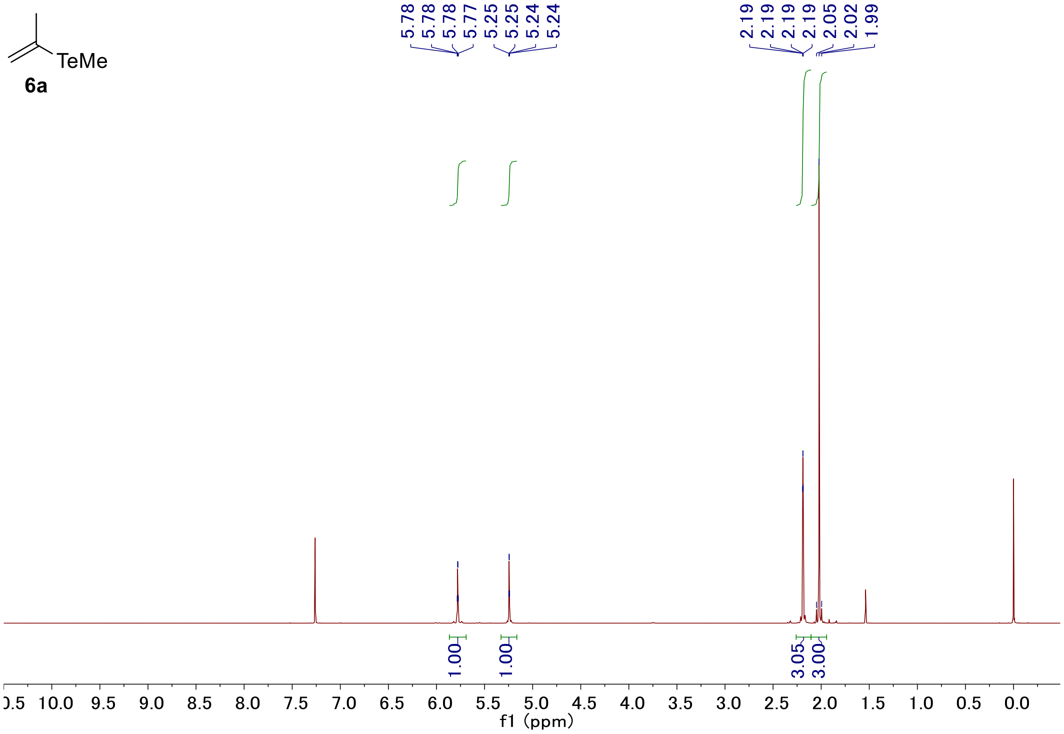

Supplementary Figure 19. ^{1}H NMR of **6a**.

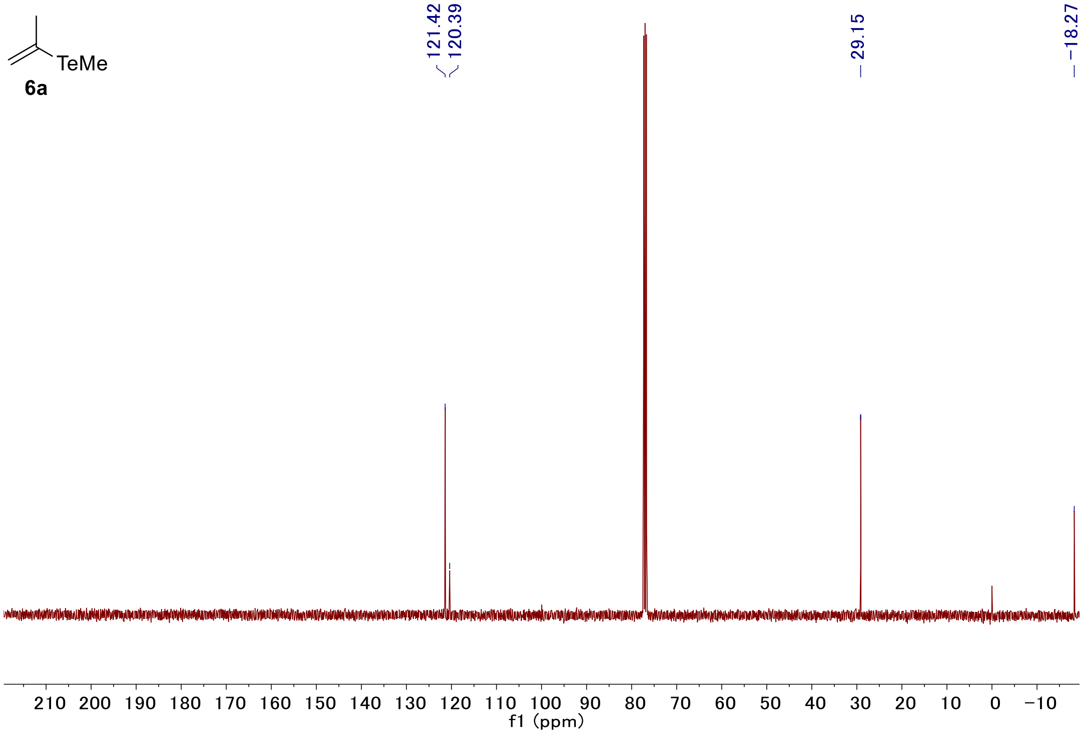

Supplementary Figure 20. ^{13}C NMR of **6a**.

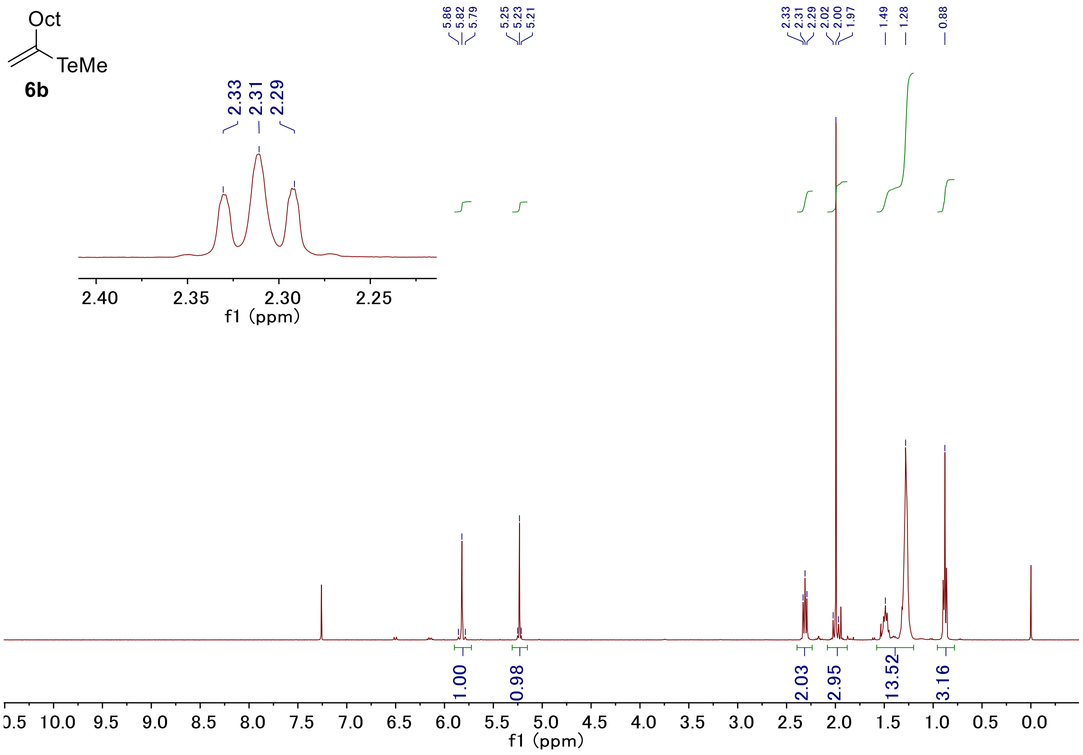

Supplementary Figure 21. ^{1}H NMR of **6b**.

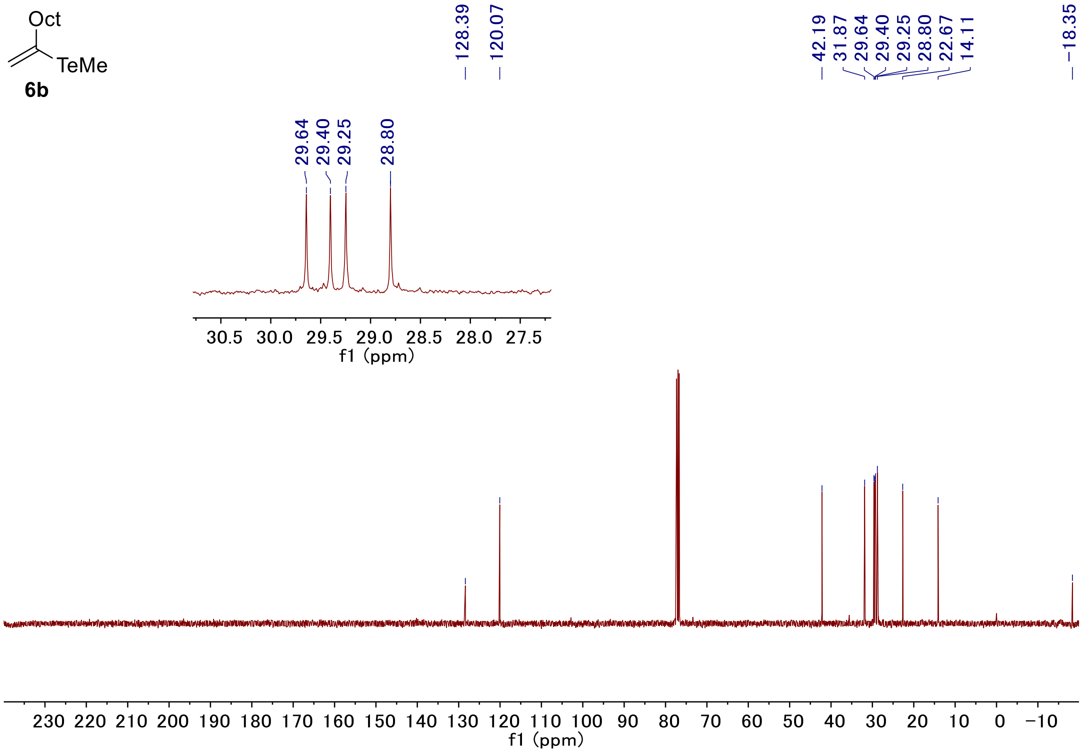

Supplementary Figure 22. ^{13}C NMR of **6b**.

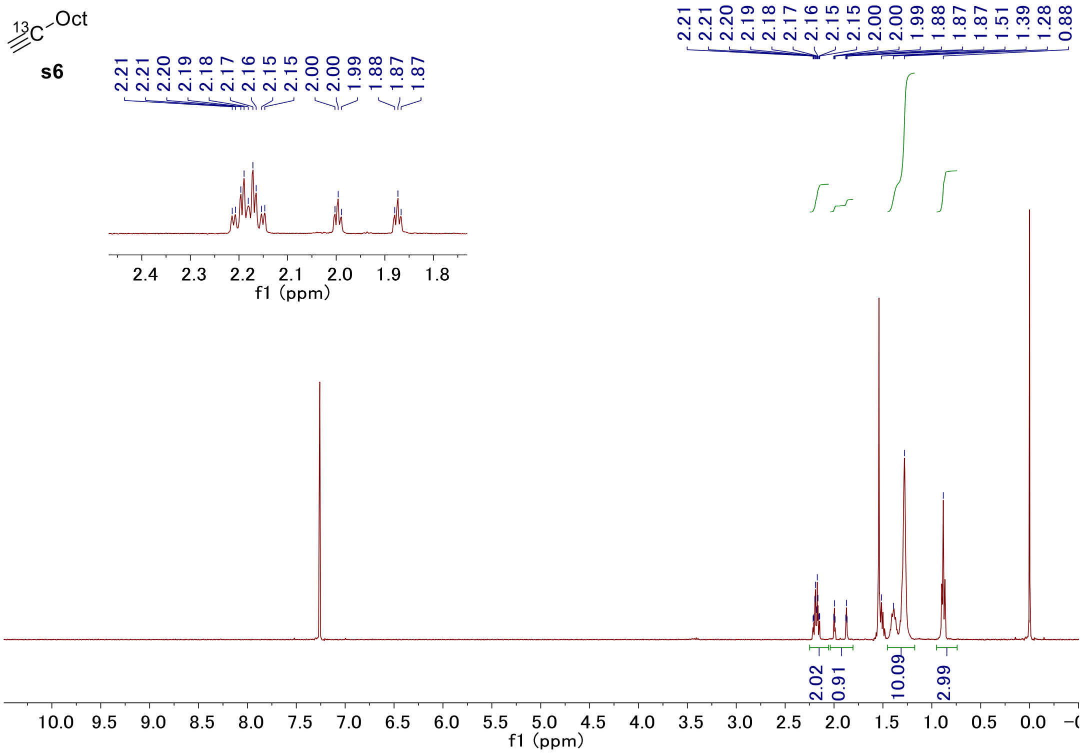

Supplementary Figure 23. ^{1}H NMR of **s6**.

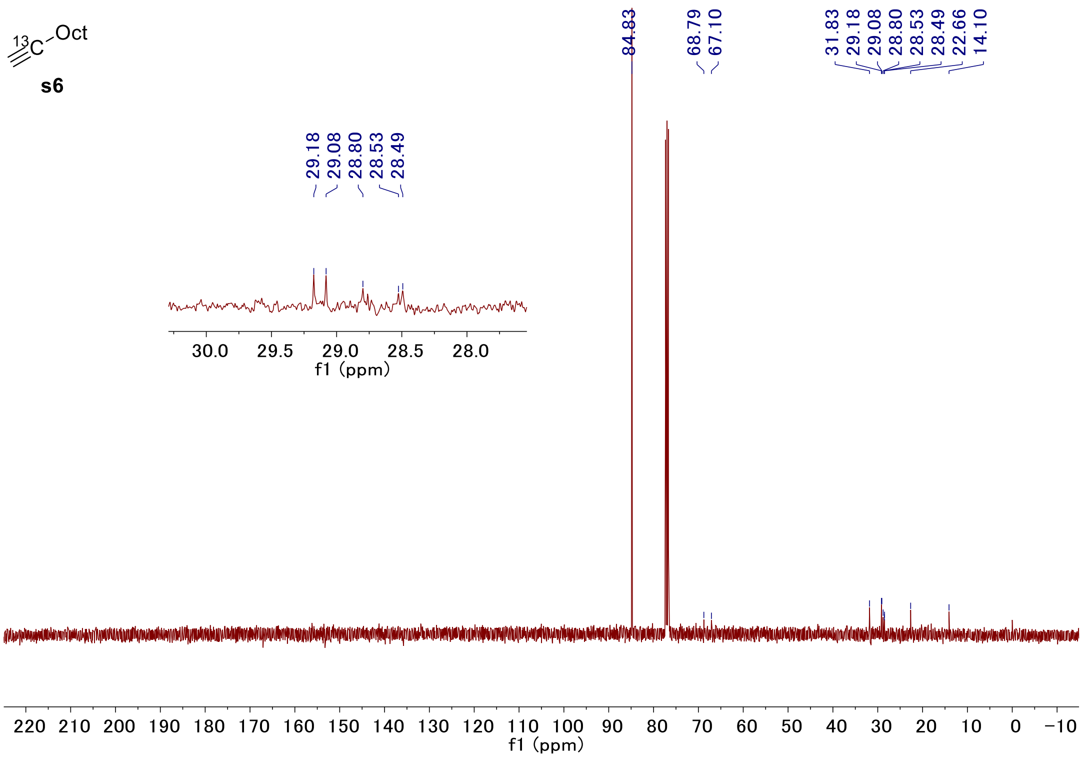

Supplementary Figure 24. ^{13}C NMR of **s6**.

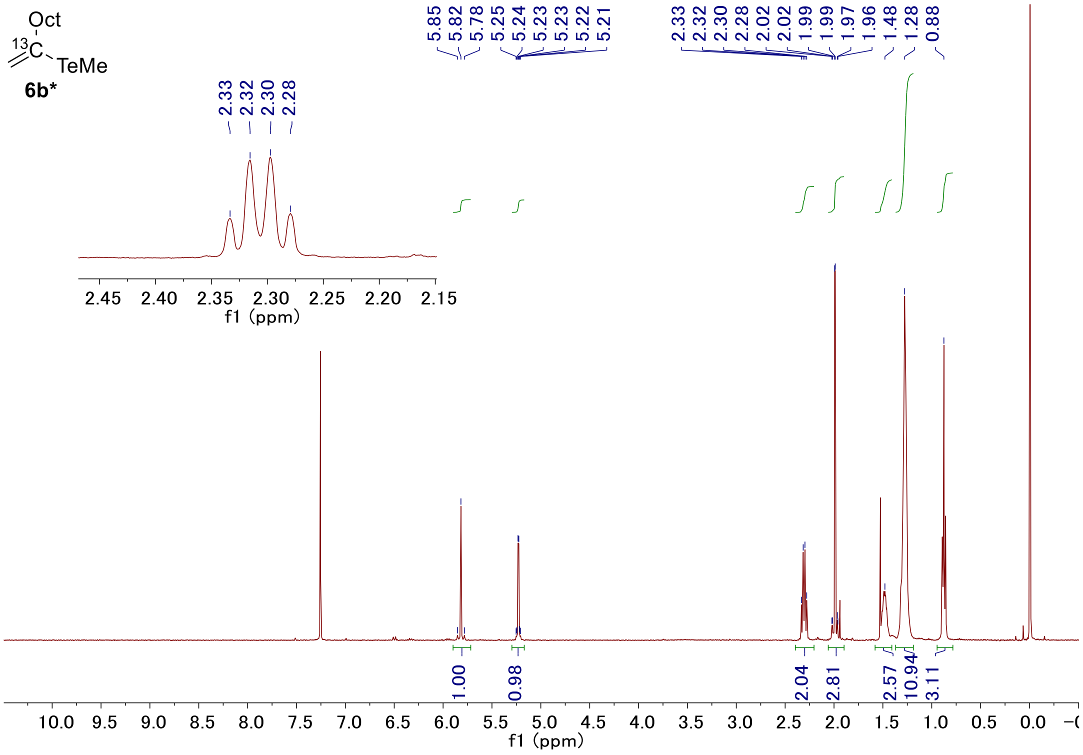

Supplementary Figure 25. ^{1}H NMR of **6b***.

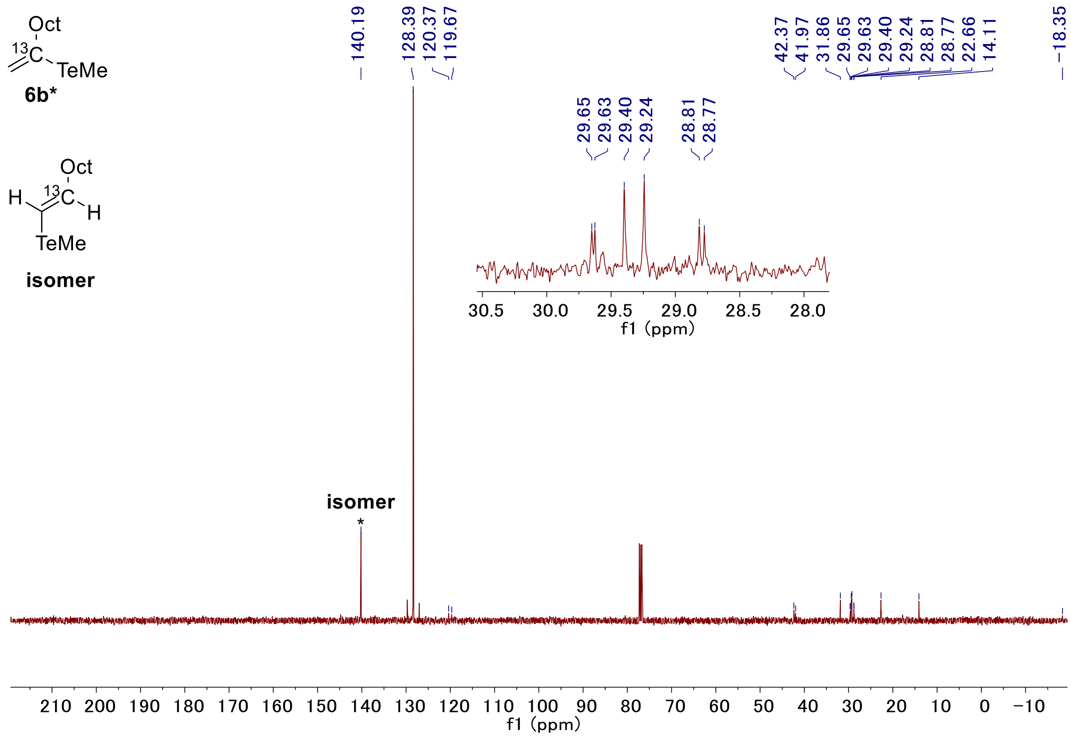

Supplementary Figure 26. ^{13}C NMR of **6b***.

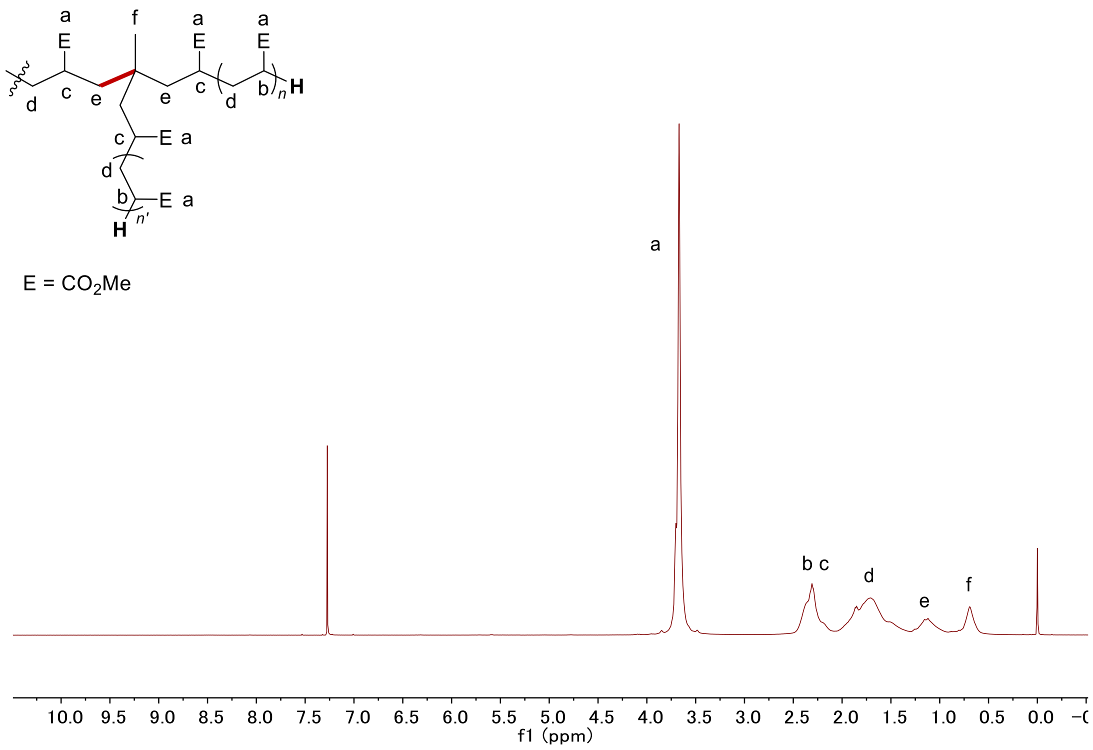

Supplementary Figure 27. ^{1}H NMR of **10d** (N = 6) prepared from **6a** and MA.

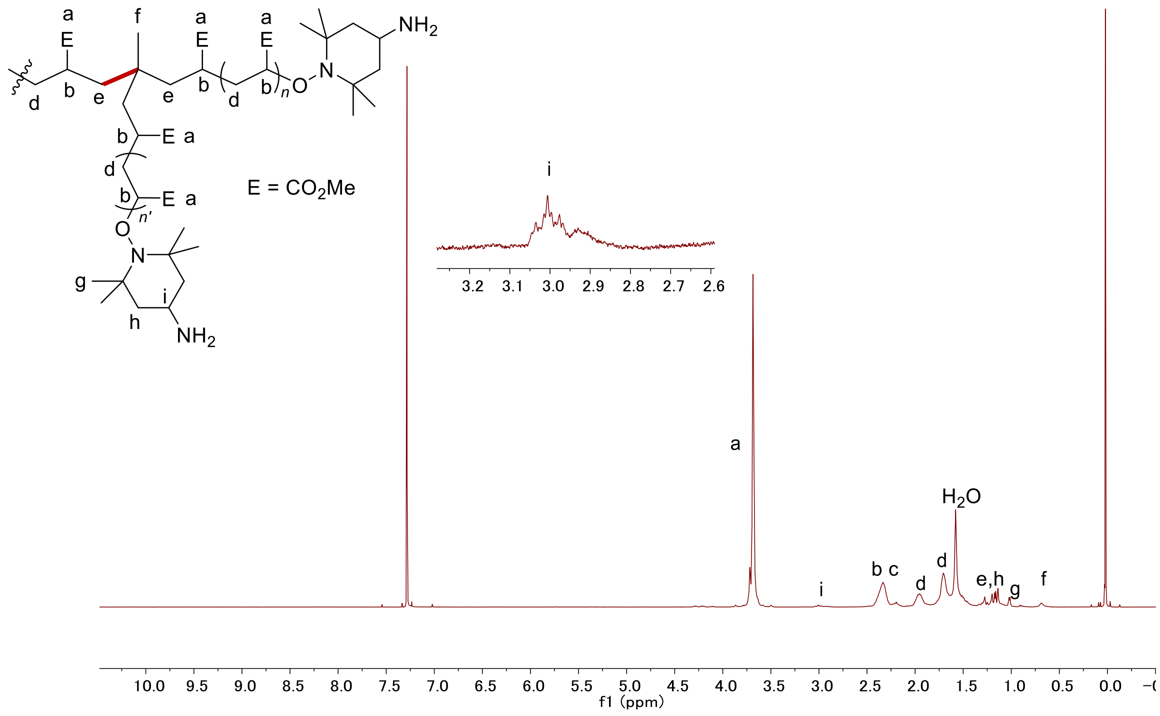

Supplementary Figure 28. ^{1}H NMR of **10e** (N = 4).

Supplementary References

1 Gaussian 09 v. Gaussian 09 (Gaussian, Inc., Wallingford, CT, 2009).

2 Yamago, S., Iida, K. & Yoshida, J. Organotellurium Compounds as Novel Initiators for Controlled/Living Radical Polymerizations. Synthesis of Functionalized Polystyrenes and End-Group Modifications. *J. Am. Chem. Soc.* **124**, 2874-2875 (2002).

3 Fan, W., Nakamura, Y. & Yamago, S. Synthesis of Mulivalent Organotellurium Chain-Tranfer Agents by Post-modification and Their Applications in Living Radical Polymerization. *Chem. Eur. J.* **22**, 17006-17010 (2016).

4 Yamago, S., Iida, K. & Yoshida, J. Tailored Synthesis of Structurally Defined Polymers by Organotellurium-Mediated Living Radical Polymerization (TERP): Synthesis of Poly(meth)acrylate Derivatives and Their Di- and Triblock Copolymers. *J. Am. Chem. Soc.* **124**, 13666-13667 (2002).

5 Drake, J. E. & Hemmings, R. T. Studies of Silyl and Germyl Group 6 Species. 5. Silyl and Germyl Derivatives of Methane- and Benzenetellurols. *Inrog. Chem.* **19**, 1879-1883 (1980).

6 Szesztay, M. & Tüdõs, F. GPC Calibration for Poly(Methyl Acrylate). *Polym. Bull.* **5**, 429-435 (1981).

7 Stickler, M., Panke, D. & Wunderlich, W. Solution properties of poly(methyl methacrylate) in methyl methacrylate, 1 Viscosities from the dilute to the concentrated solution regime. *Makromol. Chem.* **188**, 2651-2664 (1987).

8 Xie, J. Viscometric constants for small polystyrenes and polyisobutenes by gel permeation chromatography. *Polymer* **35**, 2385-2389 (1994).
